# Ewing Sarcoma of the External Ear Canal

**DOI:** 10.1155/2016/6925234

**Published:** 2016-05-30

**Authors:** Adem Binnetoglu, Tekin Baglam, Gulnur Tokuc, Kiymet Kecelioglu Binnetoglu, Fatma Gerin, Murat Sari

**Affiliations:** ^1^Department of Otorhinolaryngology-Head and Neck Surgery, Marmara University Pendik Training and Research Hospital, 34899 Istanbul, Turkey; ^2^Department of Pediatric Oncology Clinic, Marmara University Pendik Training and Research Hospital, 34899 Istanbul, Turkey; ^3^Department of Pediatrics, Marmara University Pendik Training and Research Hospital, 34899 Istanbul, Turkey; ^4^Department of Pathology, Marmara University Pendik Training and Research Hospital, 34899 Istanbul, Turkey

## Abstract

*Background.* Ewing sarcoma (ES) is a high-grade malignant tumor that has skeletal and extraskeletal forms and consists of small round cells. In the head and neck region, reported localization of extraskeletal ES includes the larynx, thyroid gland, submandibular gland, nasal fossa, pharynx, skin, and parotid gland, but not the external ear canal.* Methods.* We present the unique case of a 2-year-old boy with extraskeletal ES arising from the external ear canal, mimicking auricular hematoma.* Results.* Surgery was performed and a VAC/IE (vincristine, adriamycin, cyclophosphamide alternating with ifosfamide, and etoposide) regimen was used for adjuvant chemotherapy for 12 months.* Conclusion.* The clinician should consider extraskeletal ES when diagnosing tumors localized in the head and neck region because it may be manifested by a nonspecific clinical picture mimicking common otorhinolaryngologic disorders.

## 1. Introduction

Ewing sarcoma (ES) is a malignant tumor that consists of small round cells. It occurs in both skeletal and extraskeletal forms [1–3]. The former mostly occur on long bones of the extremities. Extraskeletal ES (EES), first described by Tefft and colleagues [4], usually originates in the soft tissues of the lower extremities, paravertebral region, chest wall, or retroperitoneum. Rarely, it arises in the head or neck [4–6]. To our knowledge, there are no previous reports in the English literature of it originating in the external ear canal (EEC).

## 2. Case Report

**Figure 1:**
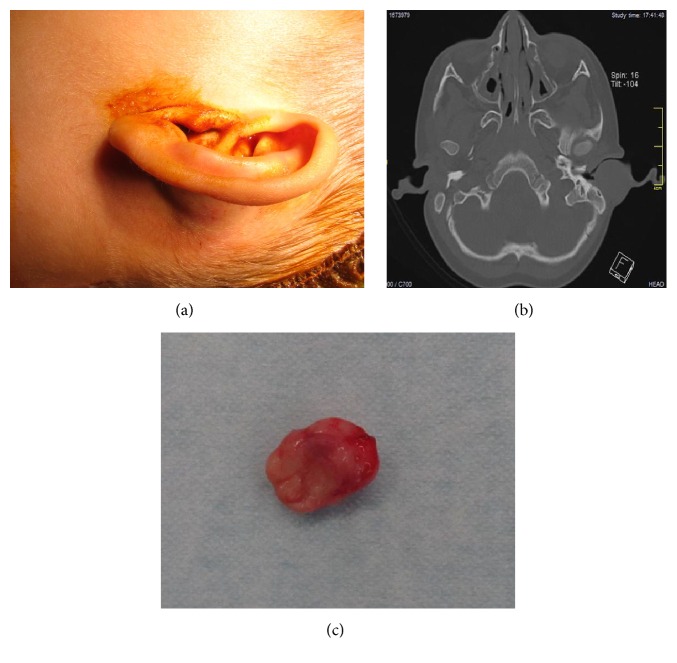
(a) Smooth, rubbery mass in the left postauricular area, displacing the conchal cartilage anteriorly and obstructing the EEC. (b) Axial CT image showing a homogenous, hypodense, solid soft tissue mass in the postauricular area. (c) Fresh, unfixed specimen after surgical removal.

A 2-year-old boy was admitted via our emergency services with a six-day history of an auricular mass which had not responded to three days of treatment with an amoxicillin clavulanate. There was no history of trauma, fever, or pain or of other features to suggest otitis media, or another upper airway infection. On physical examination, there was a painless, smooth, rubbery mass in the left postauricular area, pushing the conchal cartilage anteriorly and obstructing the EEC (Figure 1(a)). Computed tomography (CT) of the temporal bone showed a 23 × 12 mm solid soft tissue mass in the postauricular area that obstructed the EEC (Figure 1(b)). It had regular margins and was homogeneous and hypodense, compatible with an auricular hematoma. After obtaining informed consent, we explored the mass surgically in the expectation that it was likely to be an auricular hematoma. We made an incision behind the ear and elevated a skin flap. We then encountered a solid tumor, which we dissected out from surrounding tissue, obtaining a gross total resection (Figure 1(c)).

**Figure 2:**
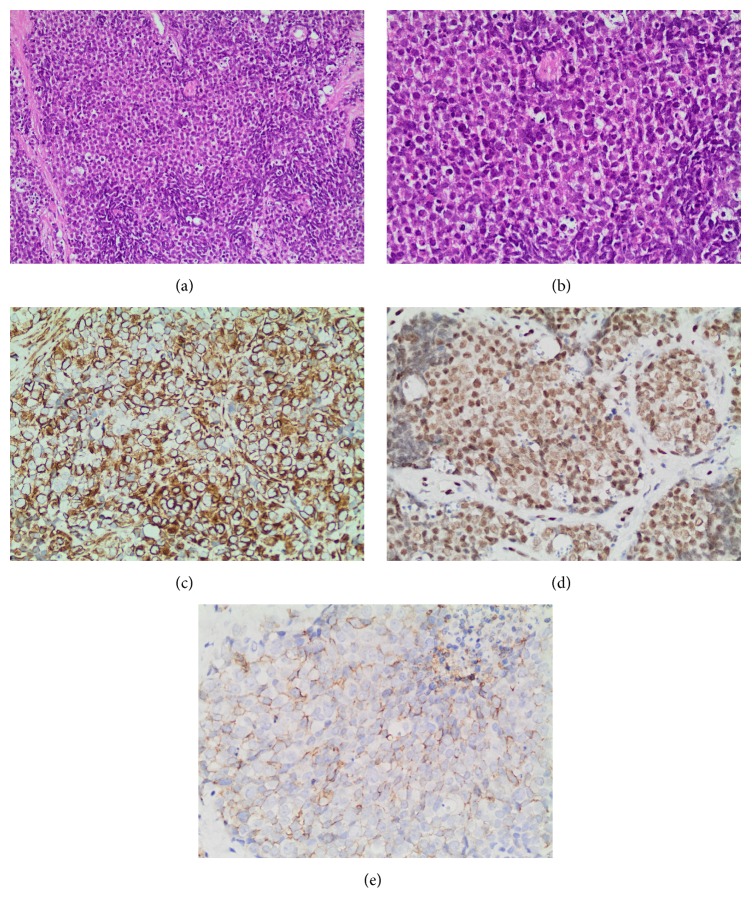
(a) Tumor cells are uniform small round cells with round nuclei containing fine chromatin, high nuclear to cytoplasmic ratio, and indistinct cytoplasmic membrane (H&E; 20x). (b) Tumor cells are uniform small round cells with round nuclei containing fine chromatin, high nuclear to cytoplasmic ratio, and indistinct cytoplasmic membrane (H&E; 40x). (c) Immunohistochemical staining for vimentin (40x). (d) Immunohistochemical staining for FLI1 (40x). (e) Immunohistochemical staining for CD99 (40x).

The tumor originated from the cartilage part of the EEC, pushing the conchal cartilage anteriorly. Pathological examination revealed a small blue round cell tumor. It was further using a panel of immunohistochemical stains. It stained negatively with markers for melanoma, lymphoma, neuroendocrine carcinomas, and rhabdomyosarcoma, but >50% of cells stained positively for CD99 and diffusely for FLI1 and vimentin. It had a high proliferative index with Ki-67 (Figure 2). Diagnosis was round cell malignant tumor consistent with ES.

Leukemia markers were negative for CD13, CD33, CD34, CD117, and MPO to rule out acute myeloid leukemia (granulocytic sarcoma).

**Figure 3:**
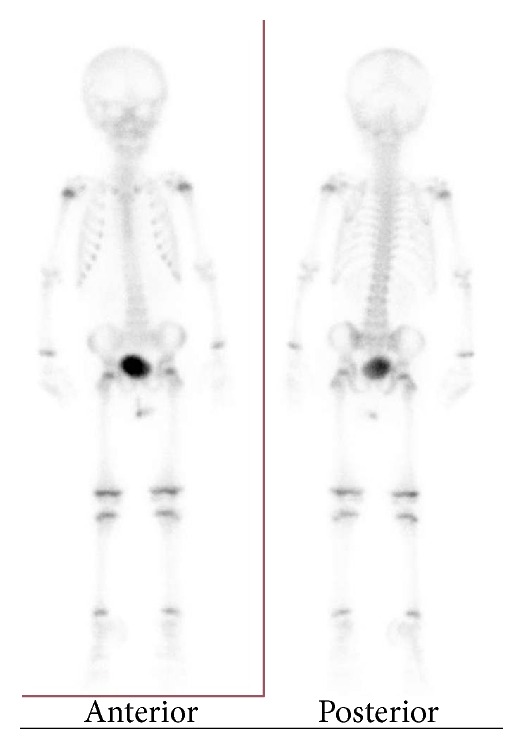
Whole body bone scan showing no bone lesions.

To exclude a metastatic tumor from an unknown primary site, whole body imaging was performed. A CT scan of the thorax, abdomen, and pelvis was normal. Cranial magnetic resonance imaging (MRI) showed only secondary intensity changes at the operation site. A radionucleotide bone scan was normal (Figure 3). The final diagnosis made was an EES originating in the EEC.

Following surgery, the patient was treated with the VAC/IE chemotherapy regimen for a year. This comprises vincristine 2 mg/m^2^, adriamycin 75 mg/m^2^, and cyclophosphamide 1200 mg/m^2^ given alternately with ifosfamide 1800 mg/m^2^/day (5 days) and etoposide 100 mg/m^2^/day (5 days) every 21 days. The treatment duration was 12 months. Given that surgery had achieved complete gross and microscopic tumor removal, radiotherapy was not given.

## 3. Discussion

EES is an uncommon tumor, accounting for 1.1% of soft tissue malignancies [2]. It usually affects males between the ages of 15 and 30 years and has an aggressive course and a high recurrence rate [7]. It may arise from various sites around the body [4–6]. It generally presents as a deep soft tissue mass without the cardinal signs of inflammation, redness, edema, and fever, but sometimes with local pain. Its imaging features are different from those of its skeletal counterpart in that it usually does not directly invade bone. Usually, it will be seen as a soft tissue mass with unclear boundaries and with hemorrhagic and/or cystic changes and areas of necrosis, but without significant calcification [8, 9]. On CT scans, it is hypodense or of mixed density, usually showing patchy contrast enhancement [9, 10]. When compared to skeletal muscle, it has low or isointense signal characteristics on T1 weighted MRI and is of high signal intensity on T2 weighted images. Again, it shows patchy contrast enhancement [11–13].

Its clinical and radiological features are nonspecific and a histological diagnosis is essential. In our patient, both the clinical and the radiological picture suggested an auricular hematoma. Following standard histopathological examination, the differential diagnosis included rhabdomyosarcoma, lymphoma, metastatic neuroblastoma, or a desmoplastic tumor. The definite diagnosis required immunohistochemical staining.

EES, when localized, is treated by surgical excision with adjuvant or neoadjuvant chemotherapy. It is responsive to a number of chemotherapeutic drugs, including vincristine, doxorubicin, and cyclophosphamide [14]. The VAC/IE regimen, as used to treat our patient, is considered standard treatment for EES [15]. Radiotherapy is used to treat localized disease which is considered inoperable, in patients with inadequate surgical margins and in those with a poor response to chemotherapy [16].

The following are reported to be good prognostic indicators: absence of metastases at presentation; tumor size less than 10 cm diameter; wide excision with clear surgical margins; chemotherapy given for eight or more cycles; a good histological response to induction chemotherapy; and combined modality treatment at presentation [17].

In conclusion, we report a unique case of EES arising from the EEC. A high index of suspicion is needed to diagnose EES, especially when they present in unusual sites such as the head or neck. Their clinical features tend to be nonspecific, and they may mimic common benign conditions, including otorhinolaryngologic disorders.
